# Supplementary material for: The Evolution and Characterization of the RNA Interference Pathways in Lophotrochozoa
Source: Genome Biol Evol. 2024 May 7;16(5):evae098. doi: 10.1093/gbe/evae098 (PMC11114477; doi:10.1093/gbe/evae098)
Supplement: evae098_Supplementary_Data [file evae098_supplementary_data.zip › Supplementary_materials.zip!/selected_miRNAs/CrGi_selected_miRNAs/CrGi_NC_047568.1_30409.pdf]

Provisional ID	:	NC_047568.1_30409
Score total	:	527.7
Score for star read(s)	:	3.9
Score for read counts	:	520.7
Score for mfe	:	2.2
Score for randfold	:	1.6
Score for cons. seed	:	-0.6
Total read count	:	1033
Mature read count	:	990
Loop read count	:	0
Star read count	:	43

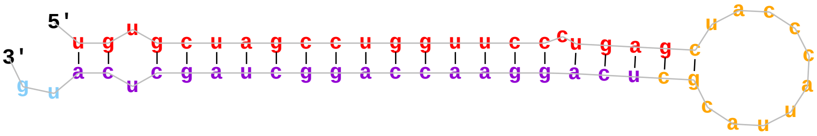

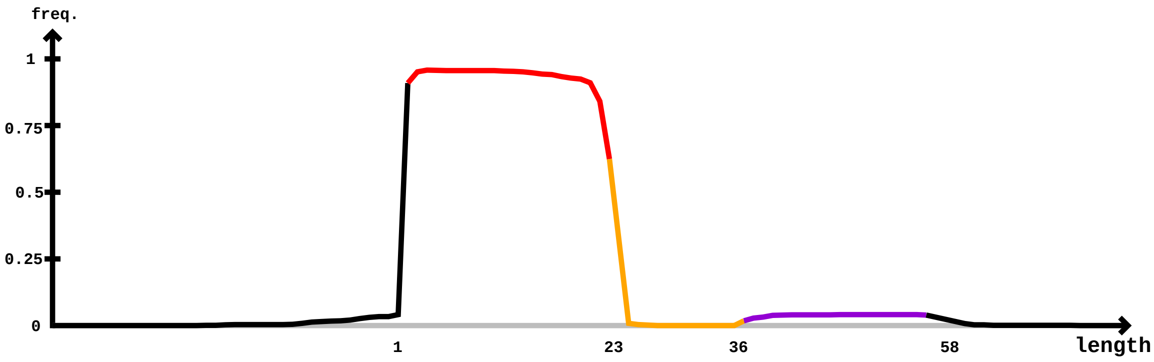

Mature Star

5'- auggcacggaaucccgaucccgauucagauaaacgugugcuagccugguucccugagcuacccauuacgcucaggaaccaggcuagcucaugcgcagauugaauuuuccc -3'	obs			
auggcacggaaucccgaucccgauucagauaaacgugugcuagccugguucccugagcuacccauuacgcucaggaaccaggcuagcucaugcgcagauugaauuuuccc	exp			
..((..(((....)))..)).(((((((.....((((.((((((((((((((.(((((..........))))))))))))))))))).))))......)))))))).....		reads	mm	sample
................aucccgauucagauaaacgugug...		1	0	Ov1
.........................cagauaaacgugugcuUgccugguucc..		1	1	Ov1
............................auaaacgugugcuagccuggu...		1	0	Ov1
.............................uaaacgugugcuagccugguucccug...		1	0	Ov1
..............................aaacgCgugcuagccugguuc...		1	1	Ov1
................................acUugugcuagccugguuc...		1	1	Ov1
................................acgugugcuagccugguuc...		1	0	Ov1
..................................Uugugcuagccugguucccugag...		1	1	Ov1
...................................ugugcuagccugguucccu..		1	0	Ov1
...................................ugugcuagccugguucccCg...		1	1	Ov1
...................................ugugcuagccugguucccug...		19	0	Ov1
...................................ugugcuaAccugguucccuga..		1	1	Ov1
...................................ugugcuagccugguucccuga..		52	0	Ov1
...................................uguUcuagccugguucccuga..		1	1	Ov1
...................................ugugcuagccCgguucccuga..		1	1	Ov1
...................................ugugcuaAccugguucccugag...		2	1	Ov1
...................................ugugcuGgccugguucccugag...		1	1	Ov1
...................................Cgugcuagccugguucccugag...		1	1	Ov1
...................................ugugcuagccugguucccugag...		117	0	Ov1
...................................Ggugcuagccugguucccugag...		1	1	Ov1
...................................ugugcuagccuAguucccugag...		2	1	Ov1
...................................ugugcuagccugguucUcugag...		1	1	Ov1
...................................ugugcuagccugguucccugCg...		1	1	Ov1
...................................ugugcuagccCgguucccugag...		2	1	Ov1
...................................ugugcuagccugguucccugagU..		2	1	Ov1
...................................ugugcuagccugguucccugagc..		7	0	Ov1
...................................ugugcuagccCgguucccugagc..		2	1	Ov1
...................................ugugcuagccAgguucccugagc..		1	1	Ov1
....................................gugcuagccugguucccug...		1	0	Ov1
....................................gugcuagccugguucccugag...		4	0	Ov1
....................................gugcuagccugguucAcugagc..		1	1	Ov1
....................................gugcuagccugguucccugagc..		2	0	Ov1
.....................................ugcuagccugguucccuga..		1	0	Ov1

```
                                      Mature                          Star
auggcacggaaucccgaucccgauucagauaaacgugugcuagccugguucccugagcuacccauuacgcucaggaaccaggcuagcucaugcgcagauugaauuuccc
.....................................ugcuagccugguucccugag....................................................   1     0     Ov1
......................................................................ucaggaaccaggcuagcuca...................   1     0     Ov1
......................................................................ucaggaGccaggcuagcuca...................   4     1     Ov1
......................................................................ucaggaGccaggcuagcucau..................   3     1     Ov1
.......................................................................caggaaccaggcuagcuca...................   1     0     Ov1
.......................................................................caggaaccaggcuagcucau..................   3     0     Ov1
.........................................................................ggaaccaggcuagcAcaug.................   1     1     Ov1
..........................................................................gaaccaggcuagcuGaugc................   1     1     Ov1

.........................cagauaaacgugugcuagcc................................................................   1     0     Ov2
.........................cagauaaacgugugcuagAcug..............................................................   1     1     Ov2
.........................cagauaaacgugugcuagccugg.............................................................   1     0     Ov2
..........................agauaaacgugugcuGgccuggu............................................................   1     1     Ov2
...........................gauaaacgugugcuagccuggu............................................................   1     0     Ov2
...............................aacgugugcuagccugguuc..........................................................   1     0     Ov2
...............................aacgugugcuagccugguucc.........................................................   1     0     Ov2
..................................gugugcuagccugguuccc........................................................   1     0     Ov2
..................................gCgugcuagccugguucccugagcua.................................................   1     1     Ov2
...................................ugugcuagccugguuccc........................................................   1     0     Ov2
...................................ugugcuagccugguucccu.......................................................   4     0     Ov2
...................................ugugcuagUcugguucccu.......................................................   1     1     Ov2
...................................ugugcuagccugguucccug......................................................   16    0     Ov2
...................................ugugcuagccugguucAcug......................................................   1     1     Ov2
...................................ugugcuagccugguucccuga.....................................................   51    0     Ov2
...................................Agugcuagccugguucccuga.....................................................   1     1     Ov2
...................................uguUcuagccugguucccuga.....................................................   1     1     Ov2
...................................ugugcuagccCgguucccuga.....................................................   3     1     Ov2
...................................ugugcuaAccugguucccugag....................................................   2     1     Ov2
...................................ugugcuagccCgguucccugag....................................................   1     1     Ov2
...................................ugugcuGgccugguucccugag....................................................   2     1     Ov2
...................................Ggugcuagccugguucccugag....................................................   1     1     Ov2
...................................ugugcuagccugguucccugag....................................................   139   0     Ov2
...................................ugugcuagccugguucccugCg....................................................   2     1     Ov2
...................................Cgugcuagccugguucccugag....................................................   1     1     Ov2
...................................ugugcuaAccugguucccugagc...................................................   1     1     Ov2
...................................ugugcuagccugguucccugagc...................................................   6     0     Ov2
....................................gugcuagccugguucccug......................................................   1     0     Ov2
....................................gugcuagccugguucccugag....................................................   5     0     Ov2
....................................gugcuagccugAuucccugagc...................................................   1     1     Ov2
....................................gugcuagccugguucccugagc...................................................   2     0     Ov2
....................................gugcuagccugguucccugagcu..................................................   1     0     Ov2
....................................gugcuagccugguucccugagcuU.................................................   1     1     Ov2
.....................................ugcuagccugguucccuga.....................................................   2     0     Ov2
.....................................ugcuagccugguucccugag....................................................   1     0     Ov2
......................................................................ucaggaaccaggcuagcuca...................   2     0     Ov2
......................................................................ucaggaGccaggcuagcuca...................   1     1     Ov2
.......................................................................caggaaccaggcuagcuc....................   2     0     Ov2
.......................................................................caggaaccaggcuagcucau..................   2     0     Ov2
........................................................................aggaaccaggcuagcucaugcg...............   1     0     Ov2
.........................................................................ggaaccaggcuagcucaugcg...............   1     0     Ov2
.........................................................................ggaaccaggcuagcucaugcgca.............   1     0     Ov2
...........................................................................aaccaggcuagcucaugcUca.............   1     1     Ov2

..............cgaucccgauucagauaaacgugu.......................................................................   1     0     Mt2
................aucccgauucagauaaacguA........................................................................   1     1     Mt2
.......................uucagauaaacgugugcuagccu...............................................................   1     0     Mt2
........................ucagauaaacUugugcuagccugg.............................................................   1     1     Mt2
........................ucagauaaacgugugUuagccuggu............................................................   1     1     Mt2
..........................agauaaaUgugugcuagccugguucc.........................................................   1     1     Mt2
..............................aaacgugugcuagAcugguu...........................................................   1     1     Mt2
..............................aaacgugugcuagAcugguuc..........................................................   1     1     Mt2
................................acgugugcuagccugguucc.........................................................   1     0     Mt2
..................................gugugcuagccugguucccug......................................................   1     0     Mt2
...................................ugugcuagccugguuccc........................................................   1     0     Mt2
...................................ugugcuagccugguuccU........................................................   1     1     Mt2
...................................ugugcuagccugguucccu.......................................................   3     0     Mt2
...................................ugugcuagccugguucccug......................................................   13    0     Mt2
...................................ugugcuagccugguucccuga.....................................................   53    0     Mt2
...................................ugugcuagccCgguucccuga.....................................................   1     1     Mt2
...................................ugugcuaAccugguucccuga.....................................................   1     1     Mt2
```

Mature **Star**

```
auggcacggaaucccgaucccgauucagauaaacgugugcuagccugguucccugagcuacccauuacgcucaggaaccaggcuagcucaugcgcagauugaauuuuccc
...................................ugugcuagccugguucccuCa......................................................  1    1  Mt2
...................................ugugcuagccCgguucccugag.....................................................  2    1  Mt2
...................................ugugcuagccugguucAcugag.....................................................  1    1  Mt2
...................................ugugcuagccugguucccugag.....................................................  136  0  Mt2
...................................ugugcuaAccugguucccugag.....................................................  2    1  Mt2
...................................ugugcuagccugguucccugCg.....................................................  1    1  Mt2
...................................uguUcuagccugguucccugag.....................................................  1    1  Mt2
...................................ugugcuagccugguucccugagc....................................................  4    0  Mt2
...................................uguUcuagccugguucccugagc....................................................  1    1  Mt2
...................................ugugcuagccCgguucccugagc....................................................  1    1  Mt2
....................................gugcuagccugguucccug.......................................................  1    0  Mt2
....................................gugcuagccugguucccuga......................................................  1    0  Mt2
....................................gugcuagccugguucccugCg.....................................................  1    1  Mt2
....................................gugcuagccugguucccugag.....................................................  7    0  Mt2
....................................gugcuagccugguucccugagc....................................................  4    0  Mt2
....................................gugcuagccugguucccugagcu...................................................  2    0  Mt2
......................................................................Ccaggaaccaggcuagcuca....................  1    1  Mt2
......................................................................ucaggaGccaggcuagcuca....................  1    1  Mt2
......................................................................ucaggaGccaggcuagcucau...................  1    1  Mt2
.......................................................................caggaaccaggcuagcucau...................  2    0  Mt2
........................................................................aggaaccaggcuagcucau...................  2    0  Mt2
.........................................................................ggaaccaggcuagcucaugcg................  2    0  Mt2
................................................................................ggcuagcucaugcgcagaAugaauu.....  1    1  Mt2

.................ucccgauucagauaGacgugugcuagcc.................................................................  1    1  Mt1
........................ucagauaaacgugugcuagccugguu............................................................  1    0  Mt1
........................ucagauaaacgugugcuagccugguuc...........................................................  1    0  Mt1
.........................cagauaaacUugugcuagccugg..............................................................  1    1  Mt1
...........................gauaaacgugugcuagccug...............................................................  1    0  Mt1
.............................uaaacgugugcuagccugg..............................................................  1    0  Mt1
.............................uGaacgugugcuagccugguucccugag.....................................................  1    1  Mt1
..............................aaacgugugcuagAcuggu.............................................................  1    1  Mt1
..............................aaacgugugcuagccugguuc...........................................................  2    0  Mt1
...............................aacgugugcuagccugguucc..........................................................  2    0  Mt1
...............................aacgugAgcuagccugguucccugagcuac.................................................  1    1  Mt1
..................................Uugugcuagccugguucccug.......................................................  2    1  Mt1
..................................gugugcuagccugguucccug.......................................................  1    0  Mt1
..................................Uugugcuagccugguucccugag.....................................................  1    1  Mt1
...................................ugugcuagccugguucccu........................................................  6    0  Mt1
...................................ugugcuagccCgguucccug.......................................................  1    1  Mt1
...................................ugugcuagccugguucccug.......................................................  13   0  Mt1
...................................ugugcuGgccugguucccug.......................................................  1    1  Mt1
...................................ugugcuagccugguuGccuga......................................................  1    1  Mt1
...................................ugugcuaAccugguucccuga......................................................  1    1  Mt1
...................................ugugcuagccugguucccuga......................................................  57   0  Mt1
...................................ugugcuagccCgguucccugag.....................................................  1    1  Mt1
...................................ugugcuUgccugguucccugag.....................................................  1    1  Mt1
...................................ugugcuagccugguucccGgag.....................................................  1    1  Mt1
...................................ugugcuagccugguucccugag.....................................................  158  0  Mt1
...................................ugugcuagccuAguucccugag.....................................................  2    1  Mt1
...................................ugugcuagccugguucccugGg.....................................................  1    1  Mt1
...................................ugugcuaAccugguucccugag.....................................................  1    1  Mt1
...................................ugugcuagccugguucccugagc....................................................  9    0  Mt1
...................................ugugcuagccugguucccugagcu...................................................  1    0  Mt1
...................................ugugcuagccugguucccugagcuaA.................................................  1    1  Mt1
....................................gugcuagccugguucccuga......................................................  2    0  Mt1
....................................gugcuagccugguucccugag.....................................................  4    0  Mt1
....................................gugcuagccugguucccugagc....................................................  3    0  Mt1
....................................gugcuagccugguucccugagcu...................................................  1    0  Mt1
.....................................ugcuagccugguucccug.......................................................  1    0  Mt1
.....................................ugcuagccugguucccugag.....................................................  1    0  Mt1
.....................................ugcuagccugguucccugagc....................................................  1    0  Mt1
......................................................................ucaggaGccaggcuagcuca....................  3    1  Mt1
......................................................................ucaggaaccaggcuagcuca....................  2    0  Mt1
.......................................................................caggaaccaggcuagcucau...................  1    0  Mt1
........................................................................aggaaccaggcuagcucau...................  1    0  Mt1
.........................................................................gUaaccaggcuagcucaugc.................  1    1  Mt1
.........................................................................ggaaccaggcuagcucaugcg................  1    0  Mt1
```
